# Role of adiponectin and free fatty acids on the association between abdominal visceral fat and insulin resistance

**DOI:** 10.1186/s12933-015-0184-5

**Published:** 2015-02-10

**Authors:** Aida Medina-Urrutia, Carlos Posadas-Romero, Rosalinda Posadas-Sánchez, Esteban Jorge-Galarza, Teresa Villarreal-Molina, María del Carmen González-Salazar, Guillermo Cardoso-Saldaña, Gilberto Vargas-Alarcón, Margarita Torres-Tamayo, Juan Gabriel Juárez-Rojas

**Affiliations:** Endocrinology Department, National Institute of Cardiology “Ignacio Chávez”, Juan Badiano No. 1 Col. Sección XVI Tlalpan, C.P, 14080 México City, México; Molecular Biology Department, National Institute of Cardiology “Ignacio Chávez”, Mexico City, Mexico; Laboratorio de Genómica de Enfermedades Cardiovasculares, Instituto Nacional de Medicina Genómica (INMEGEN), Mexico City, Mexico

**Keywords:** Adiponectin, Free fatty acids, Visceral fat, Insulin resistance

## Abstract

**Background:**

Experimental studies have shown that high free fatty acid (FFA) and low adiponectin (ADIPO) levels are involved in the mechanisms by which adiposity promotes insulin resistance (IR). However, no previous clinical studies have simultaneously analysed the relative contribution of FFA and ADIPO levels on the relation of abdominal visceral fat (AVF) with insulin resistance.

**Objective:**

To analyse the contribution of low ADIPO (adiponectin < =p25th: 8.67 μg/mL in women and 5.30 μg/mL in men), and high FFAs (FFAs > =p75th: 0.745 mEq/L in women and 0.60 mEq/L in men) to the association of high AVF (AVF > =p75th: 127 cm^2^ in women; 152.7 cm^2^ in men) with insulin resistance (HOMA-IR > =75th: 3.58 in women and 3.12 in men), in non-diabetic subjects.

**Material and methods:**

A cross-sectional analysis was performed including 1217 control participants of the Genetics of Atherosclerotic Disease study (GEA). Clinical, tomographic and biochemical parameters were measured in all participants. Logistic regression models were used to assess the association of high AVF with IR stratifying according to gender, and to normal or low ADIPO and normal or high FFA serum levels.

**Results:**

In comparison to referent group, in men low ADIPO unlike high FFA increased the risk of IR. Females with normal AVF and low ADIPO, or high AVF and normal ADIPO had aprox 3 folds risk of IR (OR [IC95%]: 3.7 [2.1-6.6], p < 0.001, and 3.4 [2.0-5.7], p < 0.001; respectively). The risk increased to 7.6 [4.2-13.8], p < 0.001 when high AVF and low ADIPO were present. Irrespective of AVF, the effect of low ADIPO on IR was higher than that seen for high FFA. Besides, our results suggest an additive effect of high AVF, high FFA and low ADIPO on the IR prevalence.

**Conclusions:**

The present study provides novel and important information about the combined effect of high AVF and low ADIPO on the risk of IR. Furthermore, our data suggest that the effect of low adiponectin levels on the high AVF-IR association is stronger than that observed for high FFA, suggesting that adiponectin could be used as biomarker to identify subjects at high risk for T2DM and CAD.

## Introduction

Coronary artery disease (CAD) and type 2 diabetes mellitus (T2DM) are currently leading causes of mortality worldwide [1]. Although obesity has been recognized as one of the most important risk factors for both these chronic diseases [2], approximately 30% of overweight or obese subjects [body mass index (BMI) ≥ 25 Kg/m^2^], do not show metabolic abnormalities [3,4]. Several studies have sought to identify when obesity is associated with metabolic abnormalities and increased risk of developing T2DM and CAD [5,6]. Abdominal visceral fat (AVF) has proven to be a better predictor of metabolic abnormalities (particularly insulin resistance) than BMI and waist circumference [7].

Experimental evidence suggests that high free fatty acid (FFA) [8] and low adiponectin (ADIPO) plasma levels [9] play a key role in the mechanisms by which excess of adiposity promotes insulin resistance [10,11]. Nevertheless, few clinical investigations have analysed the effect of high FFA serum levels on the association between increased AVF and insulin resistance (IR) [12]. As far as we know, no previous epidemiological studies have analysed the contribution of ADIPO serum levels on AVF-IR association. Considering that insulin resistance is a crucial feature of the metabolic abnormalities observed in obese subjects, the purpose of the present study was to analyse the role of ADIPO and FFA levels on the association between high AVF and IR in a Mexican-Mestizo population.

Our data suggest that the effect of low adiponectin levels on the high AVF-IR association is stronger than that observed for high FFA, suggesting that adiponectin could be used as biomarker to identify subjects at high risk for T2DM and CAD.

## Material and methods

### Study subjects

The study population was recruited from controls participating in the Genetics of Atherosclerotic Disease (GEA) study. The GEA study was designed to examine the genomic bases of CAD and to assess traditional and emerging risk factors for clinical and subclinical atherosclerotic vascular disease in the adult Mexican population [13]. All GEA participants are of self-reported Mexican-Mestizo ancestry (Spaniards and Native-American Indians). Briefly, a convenience sample of 1200 CAD patients and 1500 control subjects aged 30 to 75 years was recruited from residents in Mexico City. Patients with established premature CAD were selected from the outpatient clinic of the National Institute of Cardiology. Volunteer control participants with a negative family history of premature CAD and no personal history of cardiovascular disease were recruited from apparently healthy blood donors and through brochures posted in social service centers. Coronary patients and control subjects with history of renal, liver, thyroid or malignant disease, and corticosteroid use were not included. The GEA study was approved by the Institutional Review Board of the National Institute of Cardiology and conducted according to the Declaration of Helsinki. Written informed consent was obtained from participants.

For the purpose of the present study, we included only control subjects without T2DM. All participants answered several structured questionnaires that provide detailed information regarding family history, demographics, diet, physical activity, medications, smoking, and alcohol intake. Systolic and diastolic blood pressures were measured after subjects rest for at least 10 minutes, and the average of the second and third measurements was used as the subject’s blood pressure. Height, weight and waist circumference were measured and BMI calculated as weight in kilograms divided by height in meters squared.

### Biochemical analyses

Venous blood samples were collected from subjects after 10 hour fasting. Plasma glucose, total cholesterol, triglycerides and high density lipoprotein cholesterol (HDL-C) were measured in fresh samples, using standardized enzymatic procedures in a Hitachi 902 analyzer (Hitachi LTD, Tokyo, Japan). Accuracy and precision of lipid measurements in our laboratory are under periodic surveillance by the Centers for Disease Control and Prevention Service (Atlanta, GA). Low density lipoprotein cholesterol (LDL-C) was estimated by using the De Long et al. formula [14]. Total high sensitive C-reactive protein (hs-CRP) levels were determined by immunonephelometry on a BN ProSpec nephelometer (Dade Behring, Marburg Hesse, Germany). Inter-assay coefficient of variation was less than 6% for nephelometric determinations. Serum FFA were measured by an enzymatic-colorimetric assay (Wako Diagnostics, Chuo-Ku Osaka Japan) in a Hitachi 902 auto analyser, with intra and inter-assay variation coefficients below 3%. Total serum adiponectin was measured by Enzyme Linked Immunosorbent Assay (ELISA) technique (R & D Systems Quantikne Kit, Minneapolis, Minnesota, USA) with intra and inter-assay variation coefficients lower than 10%. Plasma insulin concentrations were determined by a radioimmunoassay (Millipore; RIA Kit, Cat. No. HI-14 K, St. Charles, Missouri, USA), the intra and inter-assay variation coefficients were 2.1% and 6.8%, respectively. Insulin resistance was estimated using the homeostasis model assessment (HOMA-IR = insulin [μIU/mL] X glucose mmol/22.5) [15]. Percentiles for insulin resistance, FFA and adiponectin levels were established in a sub-sample of the GEA control group (127 men and 169 women) without T2DM (defined as fasting plasma glucose ≥ 126 mg/dL, prior medical diagnosis, or anti-diabetic treatment use) [16], and without cardiometabolic risk factors, namely BMI < 30 kg/m^2^, blood pressure < 140/90 mmHg, fasting glucose < 100 mg/dL, HDL-C > 40 mg/dL in men and > 50 mg/dL in women, and triglyceride levels < 150 mg/dL. Insulin resistance and high FFA levels were defined as HOMA-IR and FFA levels ≥ 75th percentile (HOMA-IR: >3.58 in women and >3.12 in men; FFA levels: >0.745 mEq/L in women and >0.60 mEq/L in men), while low ADIPO was defined as serum adiponectin levels ≤ 25th percentile (≤8.67 μg/mL in women and ≤5.30 μg/mL in men).

### Computerized axial tomography study

Abdominal subcutaneous and visceral fat were quantified by computed tomography in a tomographic abdomen slice at the L4-L5 inter vertebral space. Computed tomography was performed using a 64-channel multi-detector helical computed tomography system (Somatom Sensation, Siemens. Forciem, Bavaria, Germany) and interpreted by experienced radiologists as described by Kvist *et al.* [17]. Elevated AVF was defined as AVF ≥ 75th percentile (127 cm^2^ in women; 152.7 cm^2^ in men). This cut-off point was also estimated in the sub-group of GEA participants without cardiometabolic risk factors.

### Statistical analysis

Data are presented as mean ± SD, median (interquartile range), or prevalence for categorical variables. Comparisons were made by *t* test, ANOVA and Sheffe as Post hoc, U Mann–Whitney, Kruskal-Wallis or Chi-squared test, as appropriate. In order to explore whether low ADIPO levels affect the AVF-IR association, the study population was stratified in four groups as follows: 1)Reference group: AVF < p75 (normal AVF) and adiponectin > p25 (normal ADIPO), 2) subjects with normal AVF and low ADIPO, 3)subjects with high AVF and normal ADIPO, and 4) subjects with high AVF and low ADIPO levels. To study the effect of the FFA levels on this association, the following groups were compared: 1)Reference group: normal AVF and FFA < p75 (normal FFA), 2) subjects with normal AVF and high FFA, 3) subjects with high AVF and normal FFA, and 4) subjects with high AVF and high FFA. Logistic regression models were used to analyse the effect of low ADIPO and high FFA levels on the association of high AVF with insulin resistance. Age, BMI, hs-CRP, triglycerides, HDL-C, glucose, total caloric intake, and total physical activity, were included as co-variables. The interaction of ADIPO and FFA levels with AVF was evaluated by including the AVF*FFAs or AVF*ADIPO interaction terms in the logistic regression models [18]. All p values <0.05 were considered as statistically significant. The analyses were carried out with the statistical software SPSS v15.0 (SPSS Chicago, II).

## Results

The present study included 1217 GEA control subjects (50.5% women), with a mean age of 52.5 ± 9.0 years, BMI of 28.3 ± 4.3 Kg/m^2^, and HOMA-IR median of 3.7 (2.6-5.3). Women had higher subcutaneous abdominal fat volume [320 (256–396) cm^2^ vs. 243 (183–307) cm^2^; p < 0.0001] and lower AVF volume than men [124 (93–166) cm^2^ vs. 168 (129–217) cm^2^; p < 0.0001]. Women also had higher FFA [0.61 (0.49-0.76) mEq/L vs. 0.49 (0.39-0.61) mEq/L; p < 0.0001] and higher adiponectin levels as compared to men [10.4 (6.7-15.7) μg/mL vs. 6.2 (4.0-9.4) μg/mL; p < 0.0001].

Table 1 shows the clinical characteristics of the study population stratified according to gender, normal and high AVF, and normal and low ADIPO levels. The prevalence of low ADIPO levels in individuals with normal AVF was 28.2% in women and 32.9% in men, and this prevalence was significantly higher in individuals with high AVF (40.9% in women and 44.7% in men). Normal AVF/low ADIPO individuals had significantly higher BMI and triglyceride levelsas well as lower HDL-C levels (in both genders), and significantly higher diastolic blood pressure and glucose levels (only in men) as compared to the reference group (p < 0.05). High AVF was associated with a less favorable metabolic profile in both genders, and high AVF/low ADIPO individuals showed the lowest HDL-C and the highest triglyceride concentrations among all study groups. Similarly, on stratifying according to AVF and FFA levels, high AVF/high FFA individuals showed the most unfavorable metabolic profile (data not shown), although only increased systolic blood pressure was statistically significant on comparison with the high AVF/normal FFA group, [women: 116 (108–130) mmHg vs. 111 (103–123) mmHg, p < 0.05; men: 123 (115–131) mmHg vs. 118 (111–129) mmHg, p < 0.05].

**Table 1:** **Clinical characteristics of study subjects stratified by sex, abdominal visceral fat, and adiponectin**

	**Normal AVF**		**High AVF**	
	**Normal ADIPO**	**Low ADIPO**	**Normal ADIPO**	**Low ADIPO**
**WOMEN**				
N	211	83	189	131
Age (years)	51 ± 9	49 ± 9	56 ± 8*^†^	52 ± 8^†‡^
BMI (kg/m^2^)	25(23 – 28)	26(24–29)*	30(27 – 33)*^†^	31(28–34)*^†^
SBP (mmHg)	106(97–118)	103(99–111)	113(105–124)*^†^	111(105–123)*^†^
DBP (mmHg)	67(62 – 73)	66(60–71)	71(66 – 76)*^†^	70(65–76)*^†^
LDL-C (mmol/L)	2.98 ± 0.75	3.13 ± 0.98	3.16 ± 0.83	2.98 ± 0.83
HDL-C (mmol/L)	1.47 ± 0.36	1.27 ± 0.34*	1.32 ± 0.36*	1.14 ± 0.31*^‡^
TG (mmol/L)	1.25(0.95–1.6)	1.59(1.2–2.1)*	1.65(1.3 – 2.2)*	1.76(1.4–2.3)*
Glucose (mmol/L)	4.66(4.4–4.9)	4.77(4.5–5.1)	5.05(4.7 – 5.3)*^†^	5.05(4.7–5.4)*^†^
HOMA-IR	2.5(2.0–3.4)	3.6(2.4–4.7)*	4.3(3.2 – 5.9)*^†^	5.0(3.9–6.9) *^†‡^
hs-CRP (nmol/L)	10.5(5.7–21.9)	12.4(6.7–31.4)	23.8(12.4 – 39.0)*^†^	28.6(12.4–45.7)*^†^
**MEN**				
N	157	77	204	165
Age (years)	51 ± 11	48 ± 9	55 ± 10*^†^	52 ± 8^†^
BMI (kg/m^2^)	25(23–27)	26(25–29)*	29(27 – 32)*^†^	29(27–32)*^†^
SBP (mmHg)	112(106–119)	115(107–124)	121(111–133)*^†^	119(112–127)*^†^
DBP (mmHg)	69(65–75)	72(68–76)*	75(70 – 82)*^†^	75(70–81)*^†^
LDL-C (mmol/L)	2.98 ± 0.70	3.03 ± 0.75	3.19 ± 0.85	3.16 ± 0.93
HDL-C (mmol/L)	1.19 ± 0.28	1.04 ± 0.28*	1.06 ± 0.26*	0.98 ± 0.23*^‡^
TG (mmol/L)	1.34(0.97–2.0)	1.68(1.2–2.5)*	1.90(1.5 – 2.6)*^†^	2.07(1.56–3.01)*^†‡^
Glucose (mmol/L)	4.83(4.7–5.2)	5.05(4.7–5.3)*	5.05(4.7 – 5.4)*	5.16(4.9–5.4)*^†^
HOMA-IR	2.5(1.9–3.3)	3.2(2.2–4.8)*	4.3(3.0 – 5.7)*^†^	5.1(3.4–6.7) *^†‡^
hs-CRP (nmol/L)	8.6(4.8 – 16.2)	9.5(5.7 – 18.1)	13.3(7.6 – 25.7)*^†^	15.2(8.6–28.6)*^†^

Mean ± SD or median (interquartil range); ANOVA or Kruskal-Wallis. *p < 0.05 vs. normal AVF/ normal ADIPO; ^†^p < 0.05 vs. normal AVF/low ADIPO; ^‡^p < 0.05 vs. high AVF/normal ADIPO. AVF = Abdominal visceral fat; ADIPO = Adiponectin; BMI = Body mass index; SBP = Systolic blood pressure; DBP = Diastolic blood pressure; LDL-C = Low density lipoprotein cholesterol cholesterol; HDL-C = High density lipoprotein cholesterol; TG = Triglycerides; hs-CRP = High sensitivity C-reactive protein.

**Figure 1:**
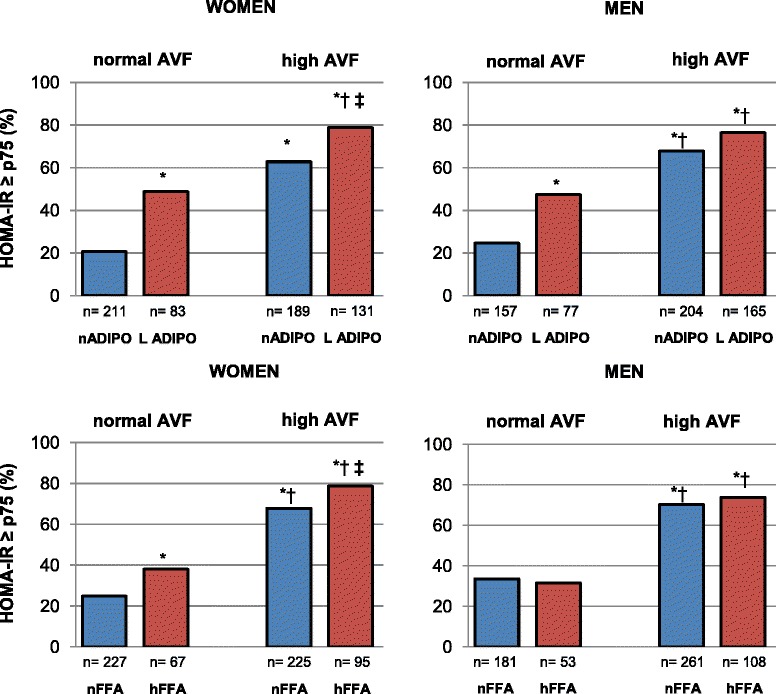
**Effect of adiponectin (ADIPO) and free fatty acids (FFA) levels on the prevalence of insulin resistance (HOMA-IR ≥ p75).** Subjects were stratified by normal or high abdominal visceral fat. *p < 0.05 as compared to normal AVF/normal ADIPO; ^†^p < 0.05 as compared to normal AVF/low ADIPO; ^‡^p < 0.05 as compared to high AVF/normal ADIPO, Chi-square test. Cut-off values are defined in material methods section.

In women, high AVF, low ADIPO and high FFA were all significantly associated with a higher prevalence of IR (Figure 1). Moreover, high AVF/low ADIPO and high AVF/high FFA combinations showed a stronger association with IR than any of these parameters assessed individually. In contrast, the prevalence of IR in men with high AVF was not significantly affected by low ADIPO or high FFA levels as assessed by univariate analysis (Figure 1), although logistic regression analyses adjusting for confounding factors, revealed that low ADIPO, but not high FFA, significantly increases the risk of IR in men with high AVF (Figure 1 and Table 2). Moreover, normal AVF/low ADIPO and high AVF/normal ADIPO women had a 3 fold increased risk of IR even after controlling for FFA levels and other confounding variables. The odds ratio for IR increased to 7.6 in the high AVF/low ADIPO group. Irrespective of the AVF, the effect of low ADIPO on IR was higher than that observed for high FFA levels. Because interactions terms AVF*ADIPO or AVF*FFA were not statistically significant in the logistic regression models, the results suggest an additive and independent effect of AVF, ADIPO and FFA levels on the prevalence of IR.

**Table 2:** Regression logistic models* analyzing the effect of AVF, adiponectin and FFA on insulin resistance prevalence

	**Normal AVF**		**High AVF**		
	**Normal ADIPO**	**Low ADIPO**	**Normal ADIPO**	**Low ADIPO**	**Interaction p**
**Women**	1	3.7(2.1-6.6)	3.4(2.0-5.7)	7.6(4.2-13.8)	0.6
**Men**	1	1.9(1.0-3.6)	2.5(1.4-4.3)	4.1(2.6-7.2)	0.7
	**Normal AVF**		**High AVF**		
	**Normal FFA**	**High FFA**	**Normal FFA**	**High FFA**	
**Women**	1	2.2(1.1-4.1)	2.8(1.7-4.6)	4.5(2.3-8.8)	0.5
**Men**	1	0.8(0.4-1.8)	2.1(1.3-3.4)	2.3(1.2-4.2)	0.6

*Age, body mass index, high sensitive C-reactive protein, physical activity, total caloric intake, and FFA or adiponectin levels were included as co-variables (similar results were obtained after including triglycerides, HDL-C and glucose plasma levels in the model). AVF = Abdominal visceral fat; FFA = Free fatty acids; ADIPO = Adiponectin; HDL-C = High density lipoprotein cholesterol. Cut-off values are defined in material method section.

## Discussion

This study shows for the first time that irrespective of AVF, low ADIPO and high FFA levels are associated with IR, and that the effect of low ADIPO is stronger than that of high FFA levels. Moreover, an additive effect of low ADIPO and high FFAs with high AVF on IR prevalence was observed. While subjects with high AVF or low ADIPO had a three-fold increased risk of IR, the combination of low ADIPO with high AVF doubled this probability. Interestingly, high FFA levels were associated with IR only in females, but were significantly associated with IR in both genders when combined with high AVF. Our results suggest that high AVF, high FFA and low ADIPO levels have an additive and independent effect increasing the risk of IR. This is particularly relevant because insulin resistance is a common alteration among obese subjects, and that both abnormalities are precursors of T2DM and CAD.

Adiponectin is a constitutively secreted adipocyte protein. In obese individuals, ADIPO serum levels are frequently low and show an inverse correlation with AVF, but not with BMI or subcutaneous fat deposits [19,20]. It has been suggested that this protein is a potent insulin-sensitizing agent [9,21]. Aguilar-Salinas *et al*. [22] reported that obese subjects with high ADIPO levels had lower insulin concentrations and a better metabolic profile, as compared to obese subjects with low ADIPO levels. Our results extend those observations, by suggesting that low ADIPO levels are associated with a higher risk of IR, independently of AVF and other confounding factors. Moreover, we found that the high AVF/low ADIPO combination has a synergic effect on IR risk. These findings are supported by *in vitro* and *in vivo* studies showing that the interaction of adiponectin with its cellular receptors (AdipoR1 and AdipoR2) activates AMP kinase promoting the translocation of GLUT4 transporters, simultaneously reduces hepatic glucose production by inhibiting the enzyme phosphoenolpyruvate carboxylase, inhibits the synthesis of fatty acids and stimulates their oxidation [21]. Adiponectin also acts as an agonist of peroxisome proliferator activated receptor (PPAR) gamma, leading additional uptake of plasma glucose [21], and enhances insulin sensitivity by increasing hepatic insulin receptor substrate 2 (IRS-2). It has been also proposed that high-molecular adiponectin binds to membrane T-cadherin of adipocytes, forming intercellular spacers and increasing the metabolic activity and insulin sensitivity of these cells [23]. Thus, all these functions confer to adponectin a key role as an insulin sensitizer in muscle, liver, and adipose tissue [21,23].

Several studies have shown that FFA levels are frequently high in obese individuals [10,11]. Excessive FFA release by AVF is particularly deleterious because it exposes the liver to increased FFA levels via portal circulation, impairing insulin liver metabolism and promoting IR [24]. However, studies analyzing the role of FFA on the association of AVF with IR are scarce. While Miller*et al*. [12] reported that FFAs do not participate in this association, our results show that women with high AVF/high FFA levels have a twofold increased risk of IR than that of women with high AVF/normal FFA levels (Table 2). The lack of association in men could be explained by the lower FFA levels observed in this gender, and because several lines of evidence show that women are more susceptible to the unfavorable metabolic effects of obesity [25]. Of note, Miller *et al*. included both men and women in his analyses, and did not analyse genders separately. Thus, gender differences may explain the discrepancies between both studies. Our results are supported by experimental studies showing a liver lipotoxic effect of high FFA [11]. High FFA impair the insulin signalling pathway by decreasing GLUT4 transporter expression [26,27], and favouring ceramide and diacylglycerol production [28].

No previous study has simultaneously analyzed the effect of low ADIPO and high FFA levels on the high AVF-IR association. The results of the present study suggest that both low ADIPO and high FFA play a role in this through independent pathways and related mechanisms. These results are supported by a previous study showing an association of adiponectin with IR, regardless of the circulating FFA [29]. Moreover, our data extend this information by showing that the effect of low ADIPO on IR is independent of AVF, and that its effect on IR is stronger than that observed for high FFA. Although these associations need to be confirmed in further studies, it is important to point out that low adiponectin concentrations, alone or combined with high AVF, could be considered a major biomarker of IR.

### Strengths and limitations

This study has several strengths. Firstly, the distribution of body fat was measured by computed tomography, which is a specific and reliable method to quantify AVF. It was thus possible to assess the role of high AVF on low ADIPO, high FFA and IR, above and beyond clinical anthropometry. Secondly, the sample size of the study population was large enough and both sexes were included; which is important because female gender has been shown to be more susceptible to unfavorable metabolic effects of obesity. Third, multivariable analyses included physical activity and caloric intake, known to affect ADIPO, FFA levels, AVF and IR. Among the study limitations, it should be mentioned that because this is across-sectional design we cannot infer causality from the results. Moreover, only total adiponectin was measured, and not its multimeric forms which are critical determinants of IR. Nevertheless, because circulating levels of high molecular adiponectin have been reported to show a strong correlation with total adiponectin circulating levels (r = 0.95, p < 0.0001) [30], the associations observed here would be expected to be similar. Insulin resistance was estimated by the HOMA-IR. This algorithm is as surrogate marker of IR, more feasible for large population studies, which correlate with the euglycemic CLAMP, the gold standard for assessing IR [31]. Finally, it should be noted that since there are no population-specific thresholds for HOMA-IR, adiponectin, and AVF, we used specific cut-off values obtained in our Mexican-Mestizo population, as Bonora *et al.* [32] and The European Group for the study of Insulin Resistance have recommended [33]. Because differences in metabolic markers measurements are well documented among different ethnic groups [34,35], these cut-off values cannot be applied to other population. Using population specific cut-off values may contribute to partly explain discrepancies in associations among different studies and populations.

## Conclusions

The results of the present study provide novel and important information about the combined effect of high AVF and low ADIPO levels on the prevalence of IR. Furthermore, our data suggest that the effect of adiponectin on the high AVF-IR association is stronger than the effect of high FFA levels. Therefore, adiponectin could be used as biomarker to identify subjects at high risk forT2DM and CAD.

## Abbreviations

CAD: Coronary artery disease
T2DM: Type 2 diabetes mellitus
BMI: Body mass index
AVF: Abdominal visceral fat
IR: Insulin resistance
GEA: Genetics of Atherosclerotic Disease
HDL-C: High density lipoprotein cholesterol
LDL-C: Low density lipoprotein cholesterol
hs-CRP: High sensitive C-reactive protein
FFAs: Free fatty acids
HOMA-IR: Homeostasis model assessment for insulin resistance
ADIPO: Adiponectin
